# Chilling susceptibility in mungbean varieties is associated with their differentially expressed genes

**DOI:** 10.1186/s40529-017-0161-2

**Published:** 2017-01-09

**Authors:** Li-Ru Chen, Chia-Yun Ko, William R. Folk, Tsai-Yun Lin

**Affiliations:** 1 Department of Horticulture and Biotechnology, Chinese Culture University, Taipei, 11114 Taiwan; 2 Institute of Plant and Microbial Biology, Academia Sinica, Taipei, 11529 Taiwan; 3 Department of Biochemistry, University of Missouri, Columbia, MO 65211 USA; 4 Department of Life Science & Institute of Bioinformatics and Structural Biology, National Tsing Hua University, Hsinchu, 30013 Taiwan

**Keywords:** Chilling regulated genes (*CORS*), Chilling tolerance, DHN, LTP, PDF, *Vigna radiata*

## Abstract

**Background:**

Mungbean (*Vigna radiata* L. Wilczek) is an economically important legume of high nutritional value, however, its cultivation is limited by susceptibility to chilling. Varieties NM94 and VC1973A, with differential susceptibility to stress, serve as good materials for uncovering how they differ in chilling tolerance. This study aimed to identify the ultrastructural, physiological and molecular changes to provide new insights on the differential susceptibility to chilling between varieties VC1973A and NM94.

**Results:**

Chilling stress caused a greater reduction in relative growth rate, a more significant decrease in maximum photochemical efficiency of PSII and DPPH scavenging activity and more-pronounced ultrastructural changes in VC1973A than in NM94 seedlings. Comparative analyses of transcriptional profiles in NM94 and VC1973A revealed that the higher expression of chilling regulated genes (*COR*s) in NM94. The transcript levels of lipid transfer protein (*LTP*), dehydrin (*DHN*) and plant defensin (*PDF*) in NM94 seedlings after 72 h at 4 °C was higher than that in its parental lines VC1973A, 6601 and VC2768A.

**Conclusions:**

Our results suggested that *LTP*, *DHN* and *PDF* may mediate chilling tolerance in NM94 seedlings.

**Electronic supplementary material:**

The online version of this article (doi:10.1186/s40529-017-0161-2) contains supplementary material, which is available to authorized users.

## Background

Mungbean (*Vigna radiata* L. Wilczek) seeds and its sprouts contain high levels of proteins rich in essential amino acids and various phytochemicals with beneficial activities, such as antioxidant, antimicrobial, anti-inflammatory, antidiabetic, antihypertensive, lipid metabolism accommodation and antitumor effects (Tang et al. 2014). According to Food and Agriculture Organization of the United Nations, worldwide production of mungbean increased fourfold to 21.4 million tons between 1990 and 2014, with the majority produced in Asia (85.4%).

Being sensitive to cold, the geographical and seasonal distribution is limited by low temperatures. Cold temperatures in the early growing season greatly affect its cultivation. Exposure of mungbean seedlings to 4 °C for 2 days resulted in irreversible cellular electrolyte leakage, but cold acclimation at 10 °C reduced the damage (Chang et al. 2001).

Chilling/cold stress limits plant growth and causes significant crop loss. Stress responses are influenced by duration of exposure (Ercoli et al. 2004), species (Carvallo et al. 2011) and the stage of plant development (Ohnishi et al. 2010). Plants native to temperate regions exhibit varying degrees of cold tolerance and can acquire freezing tolerance after cold acclimation, while many tropical and subtropical plants are sensitive to chilling at 0–15 °C (Miura and Furumoto 2013). To cope with low temperatures, plants often upregulate the expression of some cold related genes (*CORs*). At the molecular level, a C-repeat (*CRT*) or dehydration responsive element (*DRE*) is commonly found in the promoter regions of *COR*, and is bound by CRT binding factor (CBF)/DRE binding (DREB) protein (Carvallo et al. 2011). Expression of *VrDREB2A* in mungbean seedlings was markedly induced by drought, high-salt stress and ABA treatment, but only slightly by cold stress (Chen et al. 2016). Although the CBF pathway has been one of the dominant signal mechanisms mediating cold acclimation and is widely conserved in higher plants (Miura and Furumoto 2013), it may function differently in mungbean plants.

Different mungbean cultivars vary in their physio-biochemical responses to UV-B (Choudhary and Agrawal 2014), heat stress (Kaur et al. 2015) and bruchid damage (Hong et al. 2015). Genome size of different mungbean varieties ranged from 494 to 554 Mb and at least 52,739 genes have been annotated (Kang et al. 2014; Liu et al. 2016). Transcriptomic comparison between bruchid-resistant and -susceptible mungbean lines identified nucleotide variations caused by differential expressed genes and sequence-changed-protein genes of mungbean and transposon elements, besides bruchid-resistant (*Br*) genes, as putative modifier factors for bruchid resistance (Liu et al. 2016). Nevertheless, little is known about the molecular and physiological mechanisms underlying the intrinsic susceptibility of mungbean to chilling stress. We previously isolated 1198 mungbean expressed sequence tags (ESTs) informative to early seedling development and chilling response, and showed that variety NM94 maintained better membrane integrity than VC1973A under chilling/cold stress (Chen et al. 2008).

Lipid transfer proteins (LTPs) regulate diverse lipid-mediated cellular processes and accelerate transport of lipid monomers between membranes in vitro (Lev 2010). Salt and dehydration stress resulted in increased mRNA levels of mungbean VrLTPs (Liu and Lin 2003). A maize dehydrin (DHN) was found to bind to small lipid vesicles containing acidic phospholipids (Koag et al. 2003) which may stabilize lipid membranes. A broad spectrum of DHN proteins accumulate in response to chilling in *Arabidopsis*, cauliflower and yellow lupin (Rurek 2010). DHNs associate with large unilamellar vesicles emulating the lipid compositions of plasma and organelle membranes in *Thellungiella salsuginea* (Rahman et al. 2010). The mungbean VrDhn1, a Y_2_K-type DHN, can interact with DNA (Lin et al. 2012). Genes encoding plant defensins (PDFs) are up-regulated in *Oxytropis* (*Fabaceae*) species adapted to the arctic (Archambault and Strömvik 2011), and during cold acclimation in winter wheat (Gaudet et al. 2003) and *Arabidopsis* (Oono et al. 2006). Our objective was to examine how ultrastructural and physiological changes and differential gene expression of *COR*s varied between NM94 and VC1973A, and testing the hypothesis that VrLTP, VrDhn1 and PDF play important roles under chilling stress. Temporal gene expression patterns of seedlings during chilling stress were compared in order to decipher *COR*s underlying chilling tolerance.

## Methods

### Plant materials and growth conditions

Seedlings of mungbean varieties (Additional file 1: Figure S1; Shanmugasundaram et al. 2009) were prepared and the membrane damage was determined with electrolyte leakage as described in our previous report (Chen et al. 2008). At 3 days after imbibition (DAI), VC1973A and NM94 seedlings were randomly separated into two groups for cDNA microarray analysis: one group was chilled (4 °C for 72 h) then allowed to recover at 25 °C for 72 h; the other group was maintained at 25 °C as a control. Plants were harvested at 1, 2, 4, 24, 48, 72 h after chilling, and after 3 days recovery following 72 h chilling.

### Measurement of plant relative growth rate (RGR) and chlorophyll fluorescence

RGR was calculated as (ln Wt_n_ − ln Wt_0_)/t. Wt_n_ is the fresh weight of seedlings at the indicated time, Wt_0_ is the initial fresh weight of 3-DAI seedlings, and t indicates the time difference between t_n_ and t_0_. Each experiment contained five replications. Mungbean seedlings at 4 DAI were exposed to 4 °C and Fv/Fm ratios were measured at indicated times with a portable Pulse Amplitude Modulated (PAM) fluorometer (PAM 2100, Walz, Germany) after 30 min of dark adaptation. The maximum quantum efficiency of photosystem II, Fv/Fm, was calculated as (Fm − Fo)/Fm (Kitajima and Butler 1975). The injury index of Fv/Fm was defined as 100 × [1 − (Fv/Fm_t_/Fv/Fm_0_)] and electrolyte leakage as 100 × [(EC_t_ − EC_0_)/(1 − EC_0_)], in which t equals each time point and 0 equals the initial time before chilling (Eriksson et al. 2011). The correlation coefficient between the injury indices of Fv/Fm and electrolyte leakage was analyzed. The experiment included 15 replicates.

### Detection of radical scavenging activity and reactive oxygen species (ROS)

Radical scavenging activity of mungbean seedlings was measured using a modified method of Brand-Williams et al. (1995). Each mungbean seedling (~0.3 g fresh weight) was extracted with 1 ml of methanol and 50 μl of the extract was mixed with 150 μl of *1,1*-*diphenyl*-*2*-*picrylhydrazyl* (DPPH) solution for measuring the absorbance at 517 nm with a spectrophotometer (Powerwave XS2, BioTek, Bad Friedrichshall, Germany). The same volume of methanol was used as a blank. The experiment was replicated five times. DPPH scavenging activity (%) was defined as (*A*a − *A*b)/*A*a × 100%, where *A*a is the absorption of the blank; *A*b is the absorption of extract solution.

Hydrogen peroxide was detected in situ by staining with 3,3′-diaminobenzidine (DAB, Sigma-Aldrich) according to the method of Daudi et al. (2012). Mungbean seedlings at 3 DAI were treated at 4 °C and sampled at the indicated times. Each experiment contained six individual plants as biological replicates, and the experiment was duplicated.

### Electron microscopy

Leaves of 3-DAI seedlings subjected to 4 or 25 °C for 24 h were examined with transmission electron microscopy (TEM). Fixed specimens were dehydrated in a graded acetone series and embedded in Spurr’s resin according to the method of Lin et al. (2012). Sample blocks were polymerized at 70 °C for 24 h and ultrathin sections (100 nm) were mounted on 50-mesh copper grids. The ultrathin sections were stained with saturated uranyl acetate in 50% methanol for 20 min and then with 0.2% (w/v) aqueous lead citrate for 4 min. Samples were examined with a Philips CM100 BioTwin TEM (FEI Company Electron Optics, Eindhoven, Netherlands). Both number and area distribution of the vacuoles were calculated using Nikon NIS-element software.

### RNA isolation and cDNA microarray analysis

Total RNA was extracted as described in our previous study (Chen et al. 2008) and quantified at 260 nm with a NanoDrop 2000 spectrophotometer (NanoDrop Technologies, Wilmington, DE, USA). A cDNA microarray containing 735 mungbean early developmental and chilling stress-responsive uniESTs (Chen et al. 2008) was used to probe the mungbean RNAs, with *λ*DNA (TX803; Takara, Kyoto, Japan) as external and *VrActin* (accession no. AM910789) as internal controls. DNA fragments from a cDNA library of VC1973A seedlings were amplified with T7 primer (5′-TAATACGACTCACTATAGGG-3′) and reverse primer (5′-TCACACAGGAAACAGCTATGAC-3′), and those from subtractive cDNA libraries were amplified with nested primers 1 (5′-TCGAGCGGCCGCCCGGGCAGGT-3′) and 2R (5′-AGCGTGGTCGCGGCCGAGGT-3′). Purification of PCR products, microarray preparation, hybridization and data analysis were performed according to Chen et al. (2007). RNA samples of 50 μg from each time point were labeled with cyanine 3 (Cy3; control) and cyanine 5 (Cy5; 4 °C-treated plants) dyes using a 3DNA™ Array 50 Kit (Genisphere, Montvale, NJ) with 500 pg polyA^+^ λRNA (TX802; Takara, Kyoto, Japan) as an external control. The fluorescence intensity of each clone was divided by its corresponding control, and then normalized to the expression of the *VrActin* gene. Fold change for each gene was calculated by dividing the average intensity of 4 °C-treated samples by the average intensity of control samples at the corresponding time point.

### Relative quantification in real-time PCR (qRT-PCR)

qRT-PCR was performed as described in Chen et al. (2007) using primers (Additional file 2: Table S1) designed with Primer Express 2.0 Software (Applied Biosystems, Foster City, CA). The expression ratio between a target gene and the endogenous control gene *VrActin* was determined based on the 2^−∆∆Ct^ method (Livak and Schmittgen 2001).

### Statistical analysis

Data of RGR, Fv/Fm and DPPH scavenging activity were subjected to two-way analysis of variance (ANOVA) using CoStat statistical software (Cohort Berkeley, Monterey, CA). Significant differences among mungbean varieties under chilling stress were determined using Student–Newman–Keuls test.

## Results

### Chilling more profoundly decreased RGR, Fv/Fm and free radical scavenging capacity in VC1973A than in NM94

**Fig. 1:**
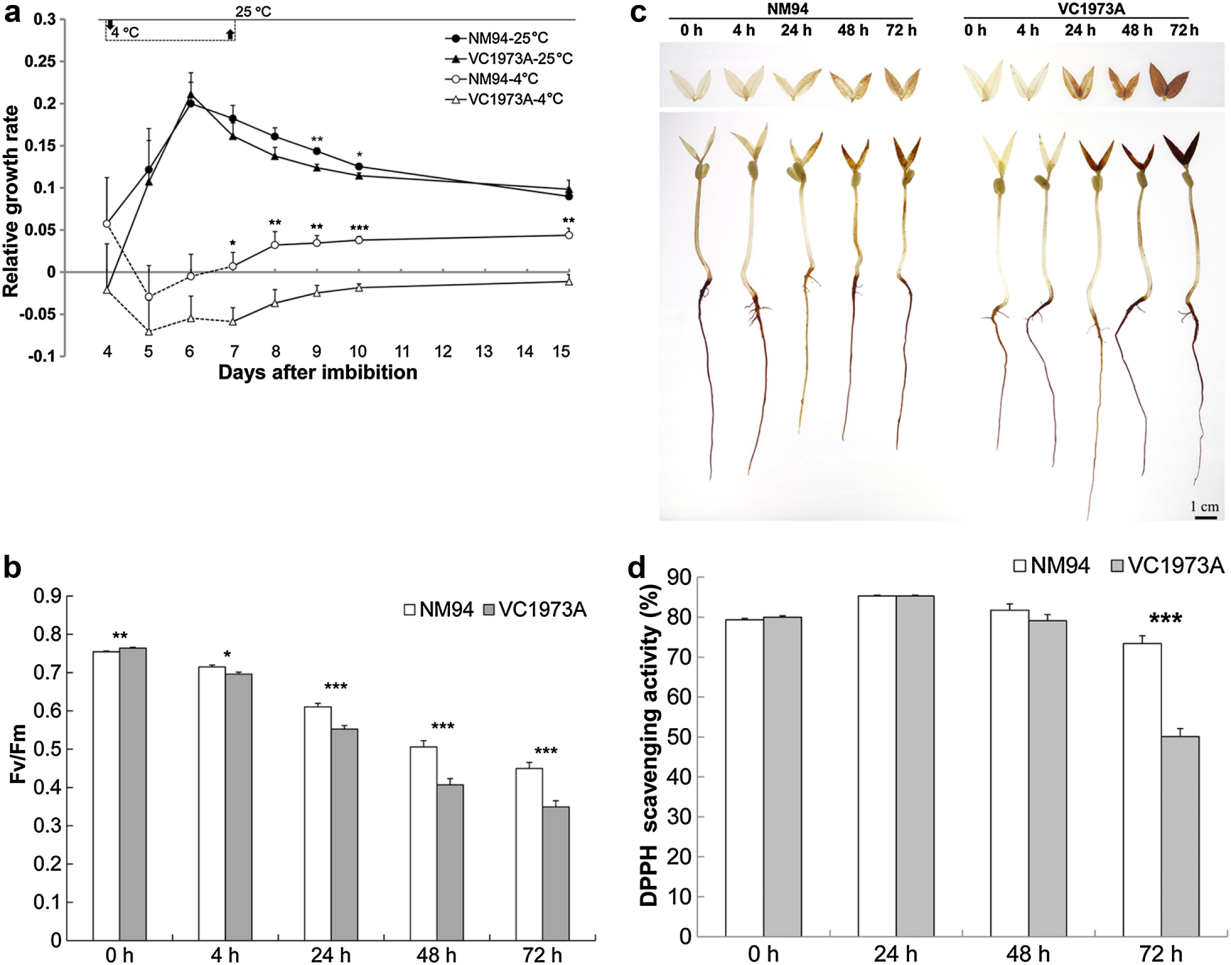
Effect of chilling/cold stress on RGR, Fv/Fm, H_2_O_2_ generation and DPPH scavenging capacity in mungbean. Leaves of 4-DAI seedling were used to measure RGR (**a**), Fv/Fm (**b**) and whole seedlings of 3-DAI were used for in situ H_2_O_2_ detection (**c**) and measurement of DPPH scavenging capacity (**d**). Treatment time in **a** is indicated by a *dash line* with starting (*downward*) and ending (*upward*) *arrows*. Data were analyzed with two-way ANOVA and Student–Newman–Keuls test. *Symbols* indicate significant difference between NM94 and VC1973A; *P < 0.05, **P < 0.01, ***P < 0.001

When grown at 25 °C, NM94 and VC1973A did not differ in RGR, and both reached a growth plateau before 6 DAI. NM94 showed more positive RGR values than VC1973A at 9–10 DAI (*P* < 0.05). Seedling growth was arrested by chilling after exposure to 4 °C for 1 day, as indicated by a negative RGR during 5–7 DAI (Fig. 1a). NM94 seedlings resumed growth after recovery from chilling, displaying positive RGR values during 7–10 DAI, while the RGR of VC1973A remained negative (*P* < 0.01).

Exposure to 4 °C for 72 h reduced Fv/Fm to 0.35 in VC1973A and 0.45 in NM94 (*P* < 0.001) from 0.75 to 0.76, respectively (Fig. 1b), showing a more severe decrease in the maximum photochemical efficiency of PSII in VC1973A. The injury index calculated from Fv/Fm values positively correlated (r^2^ = 0.98) with the electrolyte leakage in our previous study (Chen et al. 2008). Visible DAB polymerization appeared in leaves after 24–72 h chilling, indicating a stronger H_2_O_2_-dependent DAB reaction in leaves of VC1973A than NM94 under stress (Fig. 1c). Accumulation of H_2_O_2_ in roots may not be affected by chilling, as H_2_O_2_ was generated in mungbean roots without chilling. At 25 °C, VC1973A and NM94 showed a high DPPH scavenging activity of 81 and 79%, respectively. No significant difference was detected after exposure to 4 °C for 48 h (Fig. 1d). DPPH scavenging activity decreased after 72 h-chilling, but was higher in NM94 (73%) than VC1973A (50%) (Fig. 1d, *P* < 0.001), indicating a better maintenance of redox homeostasis in NM94.

### Chilling damaged the photosynthetic apparatus in mungbean seedlings

**Fig. 2:**
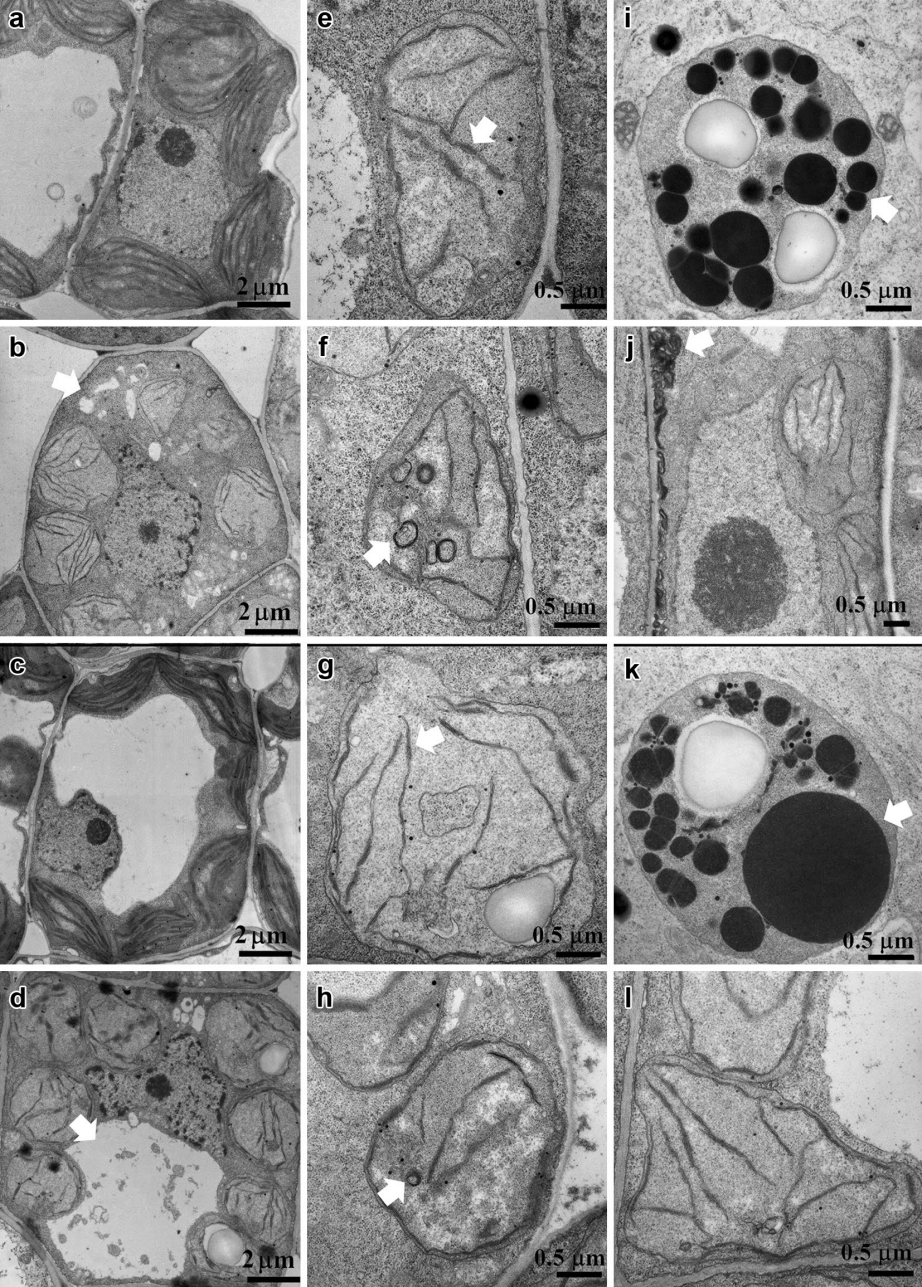
Ultrastructural changes in mesophylls and chloroplasts under chilling. 3-DAI mungbean seedlings grown at 25 °C were exposed to 25 °C (**a**, **c**) or 4 °C (**b**, **d**–**l**) for 1 day to examine ultrastructural changes (shown by *white arrows*) in VC1973A (*upper panel*) and NM94 (*lower panel*) with TEM. **a**–**d** palisade cells; **e**–**l** chloroplasts

VC1973A (Fig. 2a) and NM94 (Fig. 2c) seedlings at 3-DAI contained intact chloroplasts and well-developed granal stacks at 25 °C. Exposure to 4 °C for 1 day resulted in fragmentation of the large central vacuole in both VC1973A (Fig. 2b) and NM94 (Fig. 2d). Percentage of the cell space occupied by vacuoles in palisade mesophylls of VC1973A and NM94 at 4 °C decreased to 20.6 and 26.8%, respectively (Additional file 3: Table S2). Yet vacuoles collapsed in palisade mesophylls of VC1973A (28.6%) more than in NM94 (9.7%). Chilling also caused grana unstacking and disintegration of thylakoid membranes in spongy mesophyll of VC1973A (Fig. 2e) and NM94 (Fig. 2g). In addition, chilling triggered formation of small vesicles in chloroplast (Fig. 2f, h) and accumulation of lipid droplets (Fig. 2i, k) in both VC1973A and NM94. Importantly, chilling resulted in fractured plasma membranes in VC1973A mesophylls (21.7%, n = 60) (Fig. 2j) but not in NM94 mesophylls (Fig. 2l). This further supports our previous findings that NM94 seedlings restored plasma membrane integrity better than VC1973A (Chen et al. 2008).

### NM94 maintained a better membrane integrity than its parental lines under chilling

**Fig. 3:**
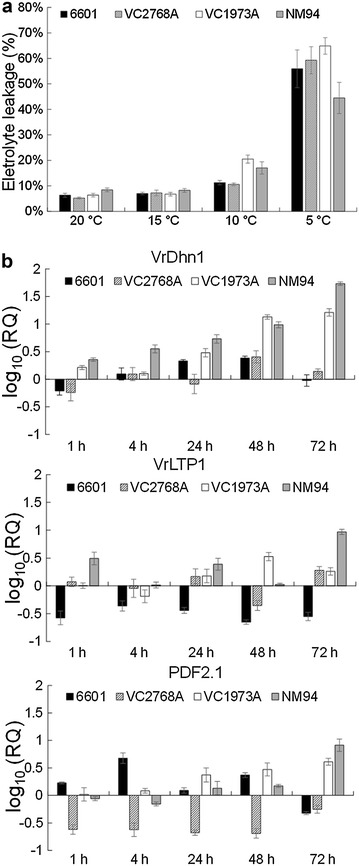
The electrolyte leakage and transcript levels of VrLTP1, VrDhn1 and PDF2.1 in mungbean seedlings under chilling. The 6601, VC2768A, VC1973A and NM94 seedling at 5-DAG were exposed to 5, 10, 15 and 20 °C for 48 h and the electrolyte leakage (**a**) were measured as the conductivity of electrolyte from leaves.* Error bar* represents standard error (n = 6). qRT-PCR (**b**) was performed to validate the relative quantification (log_10_ scale) at five time points for genes encoding VrLTP1, VrDhn1 and PDF2.1. *Error bar* represents SE

The variety NM94 has been bred by the Asian Vegetable Research and Development Center (AVRDC) through a reciprocal cross between VC1973A and a local variety 6601, and seeds of the F1 progeny were irradiated with 10 k-rad gamma rays. Up to F12, NM36 was selected for resistance to mungbean yellow mosaic virus (MYMV), and crossed with large-seeded line VC2768A to develop the MYMV-resistant and high-yield variety NM94 (Additional file 1: Figure S1). The 6601, VC2768A, VC1973A and NM94 seedling at 5-DAG were exposed to 5, 10, 15 and 20 °C for 48 h and the electrolyte leakage was examined. Leakage of electrolytes in mungbean seedlings was significantly affected by both variety and temperature based on a two-way ANOVA (Table 1). An exposure to 5 °C caused less electrolyte leakage in NM94 than that in its parental lines 6601, VC2768A, VC1973A, indicating that NM94 can maintain a better membrane integrity than its parental lines under chilling (Fig. 3a).

**Table 1:** Leakage of electrolytes in mungbean seedlings affected by variety (V) and temperature (T) based on a two-way ANOVA

Source of variance	Degree of freedom	Mean square	F value	*P* value
Variety (V)	3	123.86	2.89	0.041
Temperature (T)	3	435.10	267.10	<0.00001
V × T	9	152.19	3.55	0.0011
Error	71	42.81	–	–

### Differentially expressed *CORs* between NM94 and VC1973A seedlings

Of the 735 uniESTs examined using microarray analysis, 91 in NM94 and 40 in VC1973A were found to be *COR*s with a fold change ≥3 (up-regulated) or ≤0.3 (down-regulated) at one or more time points. Early up- (EU) or early down-regulated (ED) *COR*s were detected at 1–4 h, late up (LU) or late down (LD) *COR*s were detected at 24–72 h, and CU indicated a set of *COR*s up-regulated during 1–72 h. High correlations between qRT-PCR and microarray expression values were validated (Additional file 4: Table S3).

A venn diagram was used to compare similarities and differences between NM94 and VC1973A *COR*s gene sets, including 31 overlapping, 60 NM94 and 9 VC1973A *COR*s (Additional file 5: Table S4). NM94 *COR*s involved in photosynthesis, cellular redox homeostasis, disease resistance and membrane stabilization are listed in Table 2.

**Table 2:** Dynamic expression patterns of NM94 *COR*s under chilling stress

ID	DP^b^	NM94^a^						DP^b^	VC1973A^a^						Description
		1 h	4 h	24 h	48 h	72 h	R3d		1 h	4 h	24 h	48 h	72 h	R3d	
Photosynthesis															
MBC214	EU	2.2	*4.9*	0.9	0.5	0.5	1.4	−	1.0	1.7	0.8	0.7	1.0	1.6	Chlorophyll a/b binding protein
Contig043	EU	1.5	*4.0*	1.0	0.5	0.5	1.4	−	0.8	2.2	0.6	0.4	0.6	1.7	Chlorophyll a/b binding protein
Cellular redox homeostasis and detoxification															
VG3C101	CU	1.6	*3.5*	*5.0*	1.1	1.4	*3.1*	−	0.9	0.6	0.5	0.3	1.2	*4.2*	PDI
NG1C087	LU	1.2	1.9	2.1	2.5	*5.1*	2.4	−	1.1	0.9	1.4	1.0	2.6	*7.1*	Thioredoxin
NG3H004	CU	1.2	*11.9*	*9.1*	1.0	1.6	*7.2*	−	0.8	1.4	0.8	0.2	1.4	1.8	Glyoxalase II
Disease resistance															
Contig074	CU	*4.7*	*34.7*	*31.3*	1.4	1.7	*58.1*	−	0.4	0.9	1.0	0.0	2.7	*8.8*	PDF2.1
VG3H087	CU	2.0	*8.9*	*12.9*	0.9	1.4	*17.3*	−	1.1	0.7	0.2	0.1	2.4	*34.9*	PDF2.3
NG1C003	EU	1.5	*4.5*	2.4	0.2	0.5	*4.6*	−	0.3	0.8	0.2	0.1	0.5	*3.9*	PDF precursor
Membrane stabilization															
Contig009	CU	*4.4*	*35.4*	*34.1*	2.4	1.9	*45.9*	−	0.4	0.8	1.0	0.1	2.7	*6.9*	VrLTP1
Contig061	CU	1.1	*22.9*	*18.5*	1.1	1.8	*7.9*	−	0.5	0.7	0.6	0.1	1.0	1.2	VrDhn1

^a^Fold change of each *COR* is the ratio of average intensity of chilling-treated to average intensity of the corresponding control. The italic indicates fold change ≥3 with a *t* test *P* value <0.05

^b^‘EU’ contains genes up-regulated by chilling only in 1–4 h; ‘LU’ contains genes up-regulated only in 24–72 h; ‘CU’ indicates genes up-regulated in 1–72 h. DP, dynamic pattern. Signs of minus indicates non-*COR*s

### *COR* expression after recovery

Both varieties resumed growth (Fig. 1) and reduced electrolyte leakage after recovery at 25 °C for 3 days (Chen et al. 2008). *COR*s with more than a threefold change after recovery (R3d) were selected as the recovery responsive *COR*s (*RCOR*s), including 36 *RCOR*s in NM94 and 8 in VC1973A (Additional file 5: Table S4). NM94 and VC1973A shared 5 *RCOR*s with higher transcript levels in NM94 than VC1973A at R3d, including those encoding cysteine proteinases, proline-rich protein and peroxisomal-coenzyme A synthetase. Among the 31 NM94 *RCOR*s, the most significantly up-regulated (12- to 58-fold at R3d) genes encode ACT domain-containing protein, 8S β-globulin, VrLTP1 and PDF2.1. Transcripts of three *RCOR*s increased 3- to 11-fold at R3d in VC1973A but not NM94, one encodes ADH and two had no known homolog. Although ACT domain-containing protein, PDF2.1, PDF2.3, VrLTP1 and nuclease domain-containing protein transcripts were not significantly induced in VC1973A under cold, those transcripts (6- to 35-fold) were detected at elevated levels after recovery (Additional file 5: Table S4).

## Discussion

Low temperature at 10 °C completely suppresses chloroplast development of etiolated leaves in mungbean (cv. 2937) seedlings and severely inhibits the expression of 7 genes encoding chlorophyll a/b-binding proteins (Yang et al. 2005). In our study, NM94 seedlings maintained a more positive RGR than VC1973A at 25 °C after 6-DAI, showing a genotypic variation during seedling development. Chilling brought more damage to the photosynthetic activity (Fig. 1b) and apparatus of VC1973A (Fig. 2j) seedlings than NM94 (Fig. 2l), indicating that NM94 maintains not only better membrane integrity under chilling stress, but also maximum photochemical efficiency of PSII. Genes encoding chlorophyll a/b-binding proteins were upregulated at 4 h in NM94 under chilling (Table 1) which may contribute to better efficiency of photochemical energy conversion, similar to the finding in the desert evergreen shrub, *Ammopiptanthus mongolicus* (Wu et al. 2014).

Application of H_2_O_2_ or cold acclimation increased chilling tolerance of mungbean seedlings through ABA-independent glutathione accumulation (Yu et al. 2003) and influx of extracellular Ca^2+^ (Hung et al. 2007). NM94 seedlings under chilling stress showed less accumulation of H_2_O_2_ and higher DPPH scavenging activity than VC1973A. NM94 maintained a better balance between ROS generation and scavenging, providing greater antioxidant protection. Upon exposure to chilling, higher levels of protein disulfide isomerase (PDI) and thioredoxin transcripts accumulated in NM94 (Table 1). Early activation of the PDI gene and later activation of thioredoxin gene suggest that these enzymes cooperate to combat the oxidative stress caused by chilling. PDI reduces dehydroascorbate to l-ascorbic acid and acts against oxidative stress in chloroplasts to maintain membrane integrity and photosynthetic capacity (Huang et al. 2005). Chloroplast thioredoxins regulate photosynthetic enzymes via light-dependent reactions, playing an important role in redox regulation (Arnér and Holmgren 2000) and photosynthetic CO_2_ assimilation (Meyer et al. 2009).

A significant increase of glyoxalase II mRNA in NM94 (12-fold at 4 h, Table 1) suggests its function in detoxification of methylglyoxal (MG) which was produced under chilling stress (Hoque et al. 2012). Similarly, a rice glyoxalase II functions in salinity adaptation by maintaining better photosynthetic efficiency and anti-oxidation (Ghosh et al. 2014). Overexpression of glyoxalase pathway genes curbs the stress-induced MG level, regulates glutathione homeostasis, and helps plants survive under various abiotic stresses (Mustafiz et al. 2010).

In our experiments after 72 h at 4 °C, the *VrDhn1* mRNA level in NM94 was higher than that in VC1973A, 6601 and VC2768A (Fig. 3b; Table 1). Formation of small vesicles may increase the surface area of the chloroplast inner membrane to sustain adequate metabolite transport across the chloroplast under chilling (Kratsch and Wise 2000). We suggest that VrDhn1 may bind and stabilize small vesicles in chloroplast envelopes caused by chilling, in order to protect organelle membranes in NM94.

Transcript levels of mungbean *LTP*s were increased by salt, dehydration and exogenous ABA (Liu and Lin 2003). Interestingly, chilling resulted in rapid and marked accumulation of *VrLTP1* transcripts in NM94 but less in its parental lines VC1973A, 6601 and VC2768A (Fig. 3b; Table 1). The greater level of LTP may facilitate lipid shuttling between membranes (Douliez et al. 2000) and contribute to better membrane integrity in NM94 at 4 °C with no fractured plasma membrane in mesophyll.

NM94 has greater resistance to MYMV and cercospora leaf spot disease than VC1973A (Shanmugasundaram et al. 2009). PDFs are small, cysteine-rich peptides with antifungal, antibacterial and insect gut α-amylase inhibitory activities (Vriens et al. 2014). The transcript levels of *PDFs* are induced after 72 h at 4 °C in a greater amount in NM94 than in VC1973A, 6601 and VC2768A (Fig. 3b; Table 1). We speculate that PDFs may contribute to chilling tolerance in addition to plant defense against phytopathogenic microorganisms.

NM94 seedlings after chilling for 72 h restored growth better at 25 °C than VC1973A. The transcriptional activation of *VrDhn* and the higher levels of PDF2.1 and VrLTP1 transcripts in NM94 seedlings at R3d (Table 1) may be also involved in the recovery of growth subsequent to chilling exposure.

## Conclusions

Mungbean varieties NM94 and VC1973A possess differential susceptibility to chilling and resistance to pathogens. Chilling reduced RGR and damaged the photosynthetic apparatus in both varieties, however NM94 seedlings can sustain the maximum photochemical efficiency of PSII and DPPH scavenging activity better than VC1973A under chilling. *CORs* differentially expressed between NM94 and VC1973A upon chilling/cold stress likely play important roles in determining susceptibility to chilling. We suggested that *CORs* involved in photosynthesis, cellular redox homeostasis, disease resistance and membrane stabilization could mediate the susceptibility to chilling in NM94 seedlings. Moreover, the significant transcriptional activation of *LTP*, *DHN* and *PDFs* in NM94 seedlings under chilling may cause their greater tolerance to chilling/cold.

## Additional files

**Additional file 1: Figure S1.** The AVRDC breeding program of mungbean.

**Additional file 2: Table S1.** Oligonucleotide primers for qRT-PCR analysis.

**Additional file 3: Table S2.** Effects of chilling/cold stress on vacuoles of mesophyll cells in mungbean seedlings.

**Additional file 4: Table S3.** Validation of microarray data by qRT-PCR in mungbean seedlings.

**Additional file 5: Table S4.** Dynamic expression patterns of *CORs* in NM94 and VC1973A.

## Abbreviations

ANOVA: analysis of variance
*COR*: chilling/cold regulated gene
CRT: C-repeat
DAB: 3,3-diaminobenzidine
DAI: days after imbibition
DHN: dehydrin
DRE: dehydration responsive element
DPPH: 1,1-diphenyl-2-picrylhydrazyl
DREB: CRT binding factor (CBF)/DRE binding
ED: down-regulated regulated
EU: early up-regulated
EST: expressed sequence tag
Fv/Fm: the maximum quantum efficiency of photosystem II
LD: late down-regulated
LTP: lipid transfer protein
LU: late up-regulated
MG: methylglyoxal
PDF: plant defensin
PDI: protein disulfide isomerase
qRT-PCR: real-time PCR
RGR: relative growth rate
TEM: transmission electron microscopy
