# Supplementary material for: Chilling susceptibility in mungbean varieties is associated with their differentially expressed genes
Source: Bot Stud. 2017 Jan 9;58:7. doi: 10.1186/s40529-017-0161-2 (PMC5432936; doi:10.1186/s40529-017-0161-2)
Supplement: Supplementary file 1 — Additional file 1: Figure S1. The AVRDC breeding program of mungbean. [file 40529_2017_161_MOESM1_ESM.docx]

**Fig. S1.** The AVRDC breeding program of mungbean.

MYMV severely restricted mungbean expansion and production in South Asia. AVRDC collaborated with Nuclear Institute for Agriculture and Biology (NIAB) and the National Agricultural Research Center (NARC) in Pakistan to initiate a shuttle breeding program. In 1981, AVRDC conducted a reciprocal cross between AVRDC variety VC1973A and local variety 6601 and irradiated F1 progeny seeds with 10 k-rad gamma rays. After F2 to F12 generations of inheritance studies from 1981 to 1986, NM36 was selected for resistance to MYMV and were crossed with AVRDC large-seeded line VC2768A to develop MYMV-resistant, high-yielding mungbean variety NM94. MYMV, mungbean yellow mosaic virus; CLS, cercospora leaf spot; HR, highly resistant; R, resistant; T, tolerant; S, susceptible; HS, highly susceptible.
