# Supplementary material for: Chilling susceptibility in mungbean varieties is associated with their differentially expressed genes
Source: Bot Stud. 2017 Jan 9;58:7. doi: 10.1186/s40529-017-0161-2 (PMC5432936; doi:10.1186/s40529-017-0161-2)
Supplement: Supplementary file 2 — Additional file 2: Table S1. Oligonucleotide primers for qRT-PCR analysis. [file 40529_2017_161_MOESM2_ESM.docx]

**Table S1 Oligonucleotide primers for qRT-PCR analysis**

| **uniEST ID** | **Forward primer** | **Reverse primer** |
| --- | --- | --- |
| Contig011 | 5’-CCCCAAGAGGATCACGTCACT-3’ | 5’-CGACAGCAGCAGCACCAA-3’ |
| Contig013 | 5’-ACGGGAATTCGACCCAAAC-3’ | 5’-CCTCCACCTGGGCCTTCT-3’ |
| Contig018 | 5’-CAGAGAAGATCACAATCGACACTAAAA-3’ | 5’-CGTGGTAGGATATGGTCAGCTTT-3’ |
| Contig022 | 5'-GCGATTTCGACTTTGAGCCATG-3' | 5'-TTGCTTCCACCATCCGCAT-3' |
| Contig044 | 5’-TGGTGGTGAATTTTGGACTTATGA-3’ | 5’-CAATTTCTGCCAACCTCTTTCC-3’ |
| Contig074 | 5'-CCTTCTTGTCTTAGCCCCTGAT-3' | 5'-GTGCACAGGTGGTGTCAATTAA-3' |
| Contig076 | 5’-CTTCGCAGGGAAGCAGTTG-3’ | 5’-GGTGAAGGGTGGACTCCTTCT-3’ |
| Contig081 | 5'-ACCAAGCCATGGAGAAGAAATC-3' | 5'-CCCTGTAAGTATTCGCCAGGTT-3' |
| Contig085 | 5’-CTGCAGAGGTCGAAATGTATGTG-3’ | 5’-TCCTAGTGTTTACTGCCTGGATGTAT-3’ |
| MBA026 | 5’-GCCAGATAAGTTGGTGTCATTGTC-3’ | 5’-CCACCATTACATCCTTGGTTTTC-3’ |
| MBB009 | 5’-TTGAAAAGGCTTCTGGCAAGA-3’ | 5’-CATAAACCTCCGTGGCATCTC-3’ |
| MBB170 | 5’-CAGGGAAGTGTGGAATTGCA-3’ | 5’-CTGATAGAGACCTCATTGCTTTGG-3’ |
| MBD085 | 5’-GCAACCAAGGAGAAGCCTTCT-3’ | 5’-CTGTTGCCTTCATCGTCCAA-3’ |
| MBD127 | 5'-TGTCTTGCTGCGGTGGTAACT-3' | 5'-GCCACTCCCATGACCAAAGTCT-3' |
| MBD212 | 5’-TGCCACACCATGCCTTACC-3’ | 5’-CCACCAAGCGCCACCTT-3’ |
| MBD290 | 5’-CAGACTTTTTTCAAGATGAAGAGGATAA-3’ | 5’-TGGAAACCAAAATTACAAGGGTTT-3’ |
| MBD323 | 5’-TTCGGGAGCAAAGGATTCG-3’ | 5’-GTTCTGCCCTCGTTTCTCGTA-3’ |
| NG3C332 | 5’-GGCATTTCAGATGCACCATTTT-3’ | 5’-TTTTTCATGGAACTCTGTCAAACG-3’ |
| VG3C214 | 5’-CAGTTATCACAGGAGGCAAGTGA-3’ | 5’-GCCAAACCAACGAGCTTCTC-3’ |
| VrActin | 5'-CACCTCTGCCGAAAAGGAAAT-3' | 5'-TGACCTGTCCATCAGGCAATT-3' |
| VrDhn1 | 5'-GGCAAGTTATCAGAAGCAGTACGA-3' | 5'-GGTGACACTGGCAGCCTGAA3' |
| VrLTP1 | 5'-CCAGGCCGTGTGCAATTG-3' | 5'-TGGTGGAGGTGCTGATCTTGTA-3' |
