# Supplementary material for: Chilling susceptibility in mungbean varieties is associated with their differentially expressed genes
Source: Bot Stud. 2017 Jan 9;58:7. doi: 10.1186/s40529-017-0161-2 (PMC5432936; doi:10.1186/s40529-017-0161-2)
Supplement: Supplementary file 3 — Additional file 3: Table S2. Effects of chilling/cold stress on vacuoles of mesophyll cells in mungbean seedlings. [file 40529_2017_161_MOESM3_ESM.docx]

|  | % of the cell space  occupied by vacuole^a^ | % of cells having ruptured vacuole^b^ | Total cell numbers |
| --- | --- | --- | --- |
| 25 °C |  |  |  |
| NM94 | 41.2 ± 5.2 % | 0 % | 63 |
| VC1973A | 38.4 ± 1.6 % | 0 % | 85 |
|  |  |  |  |
| 4 °C |  |  |  |
| NM94 | 26.8 ± 1.7 % | 9.7 % | 93 |
| VC1973A | 20.6 ± 1.8 % | 28.6 % | 119 |

**Table S2 Effects of chilling/cold stress on vacuoles of mesophyll cells in mungbean seedlings**

^a^ was calculated with Nikon NIS-Element software.

^b^ was defined as vacuole disappearance. The percentage was calculated with numbers of cells having ruptured vacuoles divided with total cell number.
