# Supplementary material for: Chilling susceptibility in mungbean varieties is associated with their differentially expressed genes
Source: Bot Stud. 2017 Jan 9;58:7. doi: 10.1186/s40529-017-0161-2 (PMC5432936; doi:10.1186/s40529-017-0161-2)
Supplement: Supplementary file 4 — Additional file 4: Table S3. Validation of microarray data by qRT-PCR in mungbean seedlings. [file 40529_2017_161_MOESM4_ESM.docx]

**Table S3** Validation of microarray data by qRT-PCR in mungbean seedlings

|  | **uniEST ID** | **Fold changes in microarray data** | | | | | |  | **Fold changes in qRT-PCR** | | | | | |  | ***r*** |
| --- | --- | --- | --- | --- | --- | --- | --- | --- | --- | --- | --- | --- | --- | --- | --- | --- |
|  |  | **1 h** | **4 h** | **24 h** | **48 h** | **72 h** | **R3d** |  | **1 h** | **4 h** | **24 h** | **48 h** | **72 h** | **R3d** |  |  |
| **NM94** | |  |  |  |  |  |  |  |  |  |  |  |  |  |  |  |
|  | Contig011 | 1.3 | 1.6 | 0.6 | 0.3 | 0.3 | 1.4 |  | 1.3 | 2.1 | 0.4 | 0.1 | 0.1 | 1.1 |  | 1.0 |
|  | Contig013 | 1.2 | 1.7 | 0.5 | 0.3 | 0.2 | 1.1 |  | 1.2 | 2.0 | 0.4 | 0.2 | 0.2 | 1.5 |  | 1.0 |
|  | Contig018 | 0.7 | 1.2 | 1.6 | 1.5 | 1.7 | 2.0 |  | 0.5 | 1.1 | 1.8 | 1.3 | 1.6 | 2.2 |  | 1.0 |
|  | Contig022 | 0.9 | 1.5 | 3.6 | 6.2 | 5.7 | 0.8 |  | 0.6 | 1.0 | 6.7 | 7.8 | 20.1 | 0.8 |  | 0.8 |
|  | Contig044 | 0.8 | 0.8 | 1.3 | 0.7 | 1.0 | 7.4 |  | 0.7 | 0.5 | 2.0 | 0.3 | 0.6 | 15.4 |  | 1.0 |
|  | Contig074 | 4.4 | 32.7 | 26.7 | 1.3 | 1.6 | 51.5 |  | 1.1 | 209.9 | 212.7 | 3.9 | 2.1 | 266.1 |  | 1.0 |
|  | Contig076 | 1.1 | 1.3 | 2.5 | 2.4 | 3.6 | 1.3 |  | 0.9 | 2.1 | 3.5 | 4.9 | 4.7 | 2.7 |  | 0.9 |
|  | Contig081 | 1.8 | 11.2 | 10.6 | 0.8 | 1.4 | 19.3 |  | 2.2 | 23.8 | 26.6 | 0.5 | 1.6 | 71.0 |  | 1.0 |
|  | Contig085 | 0.9 | 1.0 | 0.5 | 0.3 | 0.4 | 1.9 |  | 0.5 | 1.2 | 0.5 | 0.3 | 0.3 | 2.0 |  | 1.0 |
|  | MBA026 | 1.1 | 1.0 | 1.1 | 1.4 | 4.4 | 17.3 |  | 1.1 | 1.0 | 1.3 | 2.1 | 5.5 | 45.5 |  | 1.0 |
|  | MBB009 | 1.3 | 1.1 | 0.5 | 0.6 | 0.9 | 13.0 |  | 1.2 | 1.2 | 0.6 | 0.4 | 0.8 | 26.9 |  | 1.0 |
|  | MBB170 | 1.2 | 1.4 | 2.0 | 1.4 | 2.8 | 9.8 |  | 0.8 | 1.6 | 2.1 | 1.7 | 2.9 | 17.6 |  | 1.0 |
|  | MBD085 | 1.4 | 1.0 | 1.2 | 2.9 | 2.5 | 1.4 |  | 0.7 | 1.0 | 1.2 | 3.2 | 2.6 | 1.9 |  | 0.9 |
|  | MBD127 | 1.3 | 1.3 | 1.3 | 0.9 | 1.4 | 2.5 |  | 0.3 | 0.3 | 1.8 | 0.7 | 1.1 | 2.6 |  | 0.8 |
|  | MBD212 | 0.8 | 0.6 | 0.9 | 0.6 | 1.0 | 2.1 |  | 0.7 | 0.6 | 0.9 | 0.4 | 0.6 | 3.3 |  | 1.0 |
|  | MBD290 | 0.7 | 0.7 | 0.6 | 0.4 | 0.4 | 1.1 |  | 0.4 | 0.7 | 0.5 | 0.3 | 0.1 | 1.3 |  | 0.9 |
|  | MBD323 | 0.8 | 0.9 | 0.5 | 0.2 | 0.3 | 0.8 |  | 0.7 | 1.1 | 0.6 | 0.2 | 0.2 | 0.8 |  | 0.9 |
|  | NG3C332 | 0.8 | 0.8 | 1.7 | 1.8 | 2.9 | 2.1 |  | 0.5 | 1.2 | 2.1 | 2.7 | 3.4 | 3.0 |  | 0.9 |
|  | VG3C214 | 1.3 | 2.0 | 0.6 | 0.4 | 0.5 | 1.5 |  | 1.5 | 3.7 | 0.8 | 0.6 | 0.5 | 2.1 |  | 1.0 |
|  | VrDhn1 | 1.1 | 22.9 | 18.5 | 1.1 | 1.8 | 7.9 |  | 0.8 | 263.4 | 139.4 | 0.7 | 4.0 | 51.7 |  | 1.0 |
|  | VrLTP1 | 4.4 | 35.4 | 34.1 | 2.4 | 1.9 | 45.9 |  | 4.2 | 33.4 | 3.5 | 0.1 | 0.1 | 8.2 |  | 0.5 |
| **VC1973A** | |  |  |  |  |  |  |  |  |  |  |  |  |  |  |  |
|  | Contig011 | 0.9 | 1.5 | 0.5 | 0.3 | 0.3 | 1.9 |  | 1.5 | 2.3 | 0.7 | 0.3 | 0.2 | 1.9 |  | 0.9 |
|  | Contig013 | 1.2 | 1.9 | 0.5 | 0.3 | 0.3 | 1.8 |  | 1.7 | 1.9 | 0.6 | 0.4 | 0.3 | 2.7 |  | 1.0 |
|  | Contig018 | 1.1 | 1.0 | 1.0 | 1.9 | 3.0 | 2.8 |  | 0.9 | 0.6 | 0.7 | 1.6 | 2.5 | 3.5 |  | 0.9 |
|  | Contig022 | 0.8 | 1.1 | 2.4 | 4.7 | 4.2 | 1.8 |  | 2.2 | 0.4 | 2.7 | 5.6 | 7.8 | 2.5 |  | 0.9 |
|  | Contig044 | 0.6 | 0.8 | 0.6 | 0.6 | 1.2 | 6.9 |  | 2.4 | 0.3 | 0.5 | 0.3 | 0.6 | 17.1 |  | 1.0 |
|  | Contig074 | 0.5 | 1.2 | 0.9 | 0.1 | 2.7 | 10.5 |  | 0.0 | 0.8 | 0.7 | 0.0 | 5.6 | 96.3 |  | 1.0 |
|  | Contig076 | 0.9 | 1.2 | 2.1 | 3.9 | 5.2 | 1.3 |  | 0.9 | 1.1 | 2.6 | 7.0 | 5.3 | 2.3 |  | 0.9 |
|  | Contig081 | 1.3 | 1.0 | 0.1 | 0.1 | 3.9 | 43.5 |  | 0.0 | 0.6 | 0.1 | 0.0 | 3.0 | 150.1 |  | 1.0 |
|  | Contig085 | 0.7 | 0.8 | 0.3 | 0.3 | 0.3 | 2.7 |  | 0.6 | 0.4 | 0.3 | 0.2 | 0.1 | 3.8 |  | 1.0 |
|  | MBA026 | 0.9 | 1.0 | 0.9 | 1.4 | 4.1 | 13.9 |  | 1.0 | 0.9 | 1.1 | 2.2 | 4.6 | 39.0 |  | 1.0 |
|  | MBB009 | 1.2 | 1.1 | 0.3 | 0.3 | 0.7 | 9.1 |  | 1.0 | 0.8 | 0.3 | 0.3 | 0.4 | 21.3 |  | 1.0 |
|  | MBB170 | 1.1 | 1.1 | 1.3 | 1.3 | 2.5 | 14.5 |  | 1.0 | 0.8 | 1.1 | 1.7 | 2.0 | 58.8 |  | 1.0 |
|  | MBD085 | 1.4 | 1.4 | 1.9 | 5.4 | 4.2 | 1.7 |  | 0.8 | 0.6 | 1.0 | 4.5 | 2.6 | 2.5 |  | 0.9 |
|  | MBD127 | 1.0 | 1.2 | 1.0 | 1.0 | 1.5 | 2.7 |  | 1.1 | 0.2 | 1.0 | 0.6 | 1.1 | 3.4 |  | 0.9 |
|  | MBD212 | 0.7 | 0.7 | 0.7 | 0.7 | 0.7 | 1.4 |  | 0.7 | 0.5 | 0.5 | 1.0 | 0.3 | 2.1 |  | 0.9 |
|  | MBD290 | 0.6 | 0.5 | 0.4 | 0.3 | 0.3 | 2.0 |  | 0.4 | 0.3 | 0.2 | 0.3 | 0.1 | 5.2 |  | 1.0 |
|  | MBD323 | 1.0 | 0.8 | 0.4 | 0.2 | 0.2 | 0.8 |  | 1.0 | 0.7 | 0.3 | 0.2 | 0.1 | 1.0 |  | 1.0 |
|  | NG3C332 | 0.7 | 0.6 | 0.8 | 1.8 | 2.8 | 2.6 |  | 0.7 | 0.5 | 0.8 | 2.0 | 2.4 | 4.9 |  | 0.8 |
|  | VG3C214 | 0.8 | 1.2 | 0.5 | 0.5 | 0.4 | 3.2 |  | 0.9 | 1.3 | 0.6 | 0.8 | 0.3 | 6.0 |  | 1.0 |
|  | VrDhn1 | 0.5 | 0.7 | 0.6 | 0.1 | 1.0 | 1.2 |  | 0.2 | 1.2 | 0.4 | 0.0 | 0.7 | 4.6 |  | 0.8 |
|  | VrLTP1 | 0.4 | 0.8 | 1.0 | 0.1 | 2.7 | 6.9 |  | 0.0 | 4.5 | 0.1 | 0.0 | 0.1 | 11.3 |  | 0.9 |

*r* indicated the correlation coefficient of microarray data and qRT-PCR data, which were calculated with relative fold changes of each selected genes at 1, 2, 4, 24, 48 and 72 h under 4°C and recovery after 72 h chilling.
