# Supplementary material for: Chilling susceptibility in mungbean varieties is associated with their differentially expressed genes
Source: Bot Stud. 2017 Jan 9;58:7. doi: 10.1186/s40529-017-0161-2 (PMC5432936; doi:10.1186/s40529-017-0161-2)
Supplement: Supplementary file 5 — Additional file 5: Table S4. Dynamic expression patterns of CORs in NM94 and VC1973A. [file 40529_2017_161_MOESM5_ESM.docx]

**Table S4 Dynamic expression patterns of *COR*s in NM94 and VC1973A**

| **ID** | | | | **DP^b^** | **NM94^a^** | | | | | |  | **DP^b^** | **VC1973A^a^** | | | | | | **Description** | |
| --- | --- | --- | --- | --- | --- | --- | --- | --- | --- | --- | --- | --- | --- | --- | --- | --- | --- | --- | --- | --- |
|  |  |  |  |  | 1 h | 4 h | 24 h | 48 h | 72 h | R3d |  |  | 1 h | 4 h | 24 h | 48 h | 72 h | R3d |  |  |
| **Common *COR*s** | | | | | | | | | | | | | | | | | | | | |
| NG1C069 | | | | CU | **4.6** | **36.7** | **33.0** | **4.2** | **3.2** | 146.0 |  | LU | 0.4 | 0.9 | 1.0 | 0.0 | **3.2** | 5.5 | no hit | |
| Contig081 | | | | CU | 2.1 | **11.3** | **12.5** | 0.8 | 1.4 | **25.4** |  | LU | 0.6 | 0.4 | 0.6 | **4.3** | **13.9** | 1.6 | PDF1 | |
| Contig049 | | | | CU | 1.9 | **3.4** | **5.7** | 1.6 | **5.0** | **5.6** |  | LU | 0.7 | 1.2 | 1.2 | 0.9 | **5.4** | 2.4 | 17.7 kDa class I sHSP | |
| NG3C013 | | | | LU | 1.2 | 2.0 | **8.5** | **5.7** | **8.1** | **4.8** |  | LU | 0.6 | 0.8 | 2.6 | **4.8** | **9.8** | 1.8 | disease resistance responsive protein | |
| NG1C247 | | | | LU | 1.5 | 2.6 | **6.6** | 1.5 | **5.6** | **3.4** |  | LU | 0.5 | 0.5 | 0.7 | 0.7 | **3.5** | 1.3 | no hit | |
| Contig002 | | | | LU | 0.9 | 1.3 | **5.7** | 0.9 | 2.2 | **11.7** |  | LU | 0.6 | 0.7 | 0.6 | 0.8 | **3.6** | **4.8** | proline-rich protein (PRP) | |
| NG1C198 | | | | LU | 1.6 | 2.2 | **5.3** | 2.5 | **4.7** | 3.2 |  | LU | 0.9 | 0.7 | 2.2 | 2.1 | **4.4** | 1.3 | nuclease HARBI1 | |
| Contig012 | | | | LU | 2.1 | 2.4 | **4.8** | 1.8 | **5.6** | **6.3** |  | LU | 0.9 | 1.1 | 1.3 | 1.6 | **5.9** | **4.2** | no hit | |
| Contig004 | | | | LU | 1.0 | 1.7 | **4.6** | **4.7** | **5.2** | 0.9 |  | LU | 0.9 | 1.1 | 2.6 | 2.9 | **3.7** | 1.6 | PRP | |
| Contig022 | | | | LU | 1.0 | 1.7 | **4.3** | **6.2** | **6.0** | 1.0 |  | LU | 0.8 | 0.9 | 2.7 | **3.9** | **4.3** | 1.4 | SAMDC | |
| MBD118 | | | | LU | 1.0 | 1.4 | **4.0** | 1.8 | 1.8 | 2.5 |  | LU | 0.9 | 0.8 | 1.4 | 2.0 | **4.8** | 1.7 | disease resistance responsive protein | |
| NG1C115 | | | | LU | 1.1 | 2.1 | **3.0** | 2.5 | 1.9 | 1.5 |  | LU | 0.8 | 1.5 | 1.7 | 1.9 | **3.0** | 1.7 | β-amylase | |
| VG3C224 | | | | LU | 0.7 | 1.1 | 1.8 | **3.9** | **8.2** | 2.1 |  | LU | 0.5 | 0.3 | 0.7 | 1.7 | **4.2** | 1.5 | peroxidase | |
| Contig135 | | | | LU | 0.9 | 0.7 | 2.0 | **3.5** | **11.6** | 56.3 |  | LU | 0.3 | 0.3 | 0.4 | 0.2 | **7.3** | 2.9 | ferredoxin-NADP^+^ reductase | |
| Contig039 | | | | LU | 1.1 | 1.0 | 2.4 | **3.3** | **7.0** | **3.3** |  | LU | 0.6 | 0.5 | 1.3 | 2.2 | **3.3** | 2.4 | nudix hydrolase | |
| Contig088 | | | | LU | 0.8 | 0.9 | 1.5 | **3.2** | **4.0** | 1.6 |  | LU | 0.6 | 0.5 | 1.1 | 2.4 | **3.3** | 1.7 | HSP70 (chloroplast) | |
| Contig019 | | | | LU | 0.9 | 2.4 | **12.9** | **9.4** | **19.1** | **8.4** |  | LU | 0.7 | 1.2 | **4.3** | **7.2** | **15.4** | **7.4** | peroxisomal-coenzyme A synthetase | |
| MBC173 | | | | LU | 1.7 | 1.9 | **6.2** | 2.5 | 1.7 | **3.1** |  | LU | 1.2 | 1.8 | **3.6** | **3.4** | **5.8** | 2.5 | chalcone synthase | |
| MBB183 | | | | LU | 1.6 | 2.3 | **4.8** | 2.5 | **3.8** | 2.0 |  | LU | 1.3 | 1.8 | **3.1** | 2.0 | 2.5 | 1.0 | FAF protein (chloroplast) | |
| Contig122 | | | | LU | 1.4 | 0.9 | **4.5** | **7.8** | **14.5** | 2.1 |  | LU | 1.3 | 1.4 | **3.6** | **7.2** | **15.1** | 0.9 | aldo/keto reductase | |
| NG3C323 | | | | LU | 1.2 | 1.5 | 2.8 | **3.3** | **3.5** | 1.8 |  | LU | 0.8 | 1.0 | 2.1 | **3.2** | **4.4** | 1.1 | no hit | |
| H088 | | | | LU | 1.3 | 1.3 | 1.4 | **3.2** | **3.1** | 2.1 |  | LU | 0.9 | 0.7 | 1.3 | **3.3** | **3.5** | 1.6 | 20 kDa chaperonin (chloroplast) | |
| VG3C251 | | | | LU | 2.3 | 2.0 | 2.5 | 1.8 | **4.9** | **16.6** |  | LU | 0.5 | 0.5 | 1.5 | 0.6 | **4.4** | 2.7 | trypsin inhibitor | |
| MBA026 | | | | LU | 1.3 | 1.1 | 1.3 | 1.4 | **4.7** | **19.9** |  | LU | 0.9 | 0.7 | 1.1 | 1.1 | **4.2** | **11.2** | cysteinyl endopeptidase | |
| MBD131 | | | | LU | 2.0 | 1.3 | 2.9 | 1.8 | **4.6** | **7.0** |  | LU | 1.0 | 0.8 | 1.4 | 2.5 | **7.3** | 2.8 | copper chaperone homolog | |
| Contig072 | | | | LU | 1.2 | 1.2 | 1.8 | 2.1 | **4.5** | 1.1 |  | LU | 0.7 | 0.5 | 0.9 | 2.5 | **5.6** | 0.6 | sHSP (chloroplast) | |
| MBC069 | | | | LU | 1.4 | 1.3 | 1.3 | 2.4 | **3.1** | 2.8 |  | LU | 1.0 | 1.0 | 1.5 | 2.6 | **4.7** | 1.7 | peptidyl-prolyl cis-trans isomerase | |
| VG3C072 | | | | LU | 1.4 | 1.7 | 2.3 | 2.2 | **3.0** | **10.8** |  | LU | 1.8 | 1.7 | 2.3 | 1.8 | **4.6** | **9.4** | cysteine proteinase | |
| MBB181 | | | | LU | 1.9 | 1.3 | 2.8 | 1.7 | **4.7** | **7.6** |  | LU | 1.0 | 0.8 | 1.5 | **3.9** | **7.8** | 2.9 | peroxidase | |
| Contig076 | | | | LU | 1.2 | 1.4 | 2.8 | 2.4 | **3.6** | 1.5 |  | LU | 0.9 | 0.9 | 2.5 | **3.1** | **5.2** | 1.0 | polyubiquitin | |
| VG3H097 | | | | CU | 1.3 | **6.8** | **5.1** | 0.7 | 0.6 | **20.5** |  | LD | 0.4 | 0.4 | 0.4 | **0.0** | 0.4 | 1.4 | no hit | |
| **NM94 *COR*s** | | | | | | | | | | | | | | | | | | | | |
| Contig127 | | | EU | | 1.8 | **5.2** | 2.7 | 0.2 | 0.5 | **4.7** |  |  | 0.3 | 0.7 | 0.2 | 0.1 | 0.3 | **3.8** | PPPDE peptidase domain-containing protein | |
| MBC214 | | | EU | | 2.2 | **4.9** | 0.9 | 0.5 | 0.5 | 1.4 |  |  | 1.0 | 1.7 | 0.8 | 0.7 | 1.0 | 1.6 | chlorophyll a/b binding protein | |
| NG1C275 | | | EU | | 1.7 | **4.8** | 2.7 | 0.2 | 0.6 | **5.3** |  |  | 0.3 | 0.8 | 0.2 | 0.1 | 0.3 | **3.7** | nucleoside-diphopshate-sugar dehydratase | |
| NG1C003 | | | EU | | 1.5 | **4.5** | 2.4 | 0.2 | 0.5 | **4.6** |  |  | 0.3 | 0.8 | 0.2 | 0.1 | 0.5 | **3.9** | PDF precursor | |
| Contig001 | | | EU | | 1.2 | **4.0** | 1.3 | 0.9 | 2.1 | **5.9** |  |  | 1.0 | 1.0 | 1.0 | 1.2 | 2.8 | **4.0** | leginsulin | |
| Contig043 | | | EU | | 1.5 | **4.0** | 1.0 | 0.5 | 0.5 | 1.4 |  |  | 0.8 | 2.2 | 0.6 | 0.4 | 0.6 | 1.7 | chlorophyll a/b binding protein | |
| MBC001 | | | EU | | 2.1 | **3.6** | 0.6 | 0.4 | 0.4 | 1.6 |  |  | 1.1 | 1.9 | 0.8 | 0.6 | 0.7 | 2.0 | serine/threonine protein phosphatase | |
| MBC024 | | | EU | | 1.6 | **3.4** | 1.6 | 0.7 | 0.7 | 1.4 |  |  | 1.0 | 2.0 | 1.1 | 0.5 | 0.4 | 1.1 | glyceraldehyde-3-phosphate dehydrogenase | |
| Contig074 | | | CU | | **4.7** | **34.7** | **31.3** | 1.4 | 1.7 | **58.1** |  |  | 0.4 | 0.9 | 1.0 | 0.0 | 2.7 | **8.8** | PDF2.1 | |
| VG3H117 | | | CU | | **4.5** | **30.7** | **29.3** | 1.5 | 1.9 | **24.0** |  |  | 0.5 | 0.8 | 1.0 | 0.0 | 2.1 | **5.8** | ACT domain-containing protein | |
| Contig009 | | | CU | | **4.4** | **35.4** | **34.1** | 2.4 | 1.9 | **45.9** |  |  | 0.4 | 0.8 | 1.0 | 0.1 | 2.7 | **6.9** | VrLTP1 | |
| NG1C004 | | | CU | | **4.2** | **30.2** | **28.7** | **3.0** | 1.9 | 39.0 |  |  | 0.5 | 0.8 | 0.9 | 0.0 | 1.7 | 4.6 | no hit | |
| NG1C195 | | | CU | | **3.9** | **39.5** | **37.7** | 1.7 | 1.9 | - |  |  | 0.3 | 0.7 | 0.7 | 0.0 | 1.6 | **8.9** | no hit | |
| VG1C030 | | | CU | | **3.7** | **65.6** | **28.6** | 3.6 | 1.2 | - |  |  | 0.47 | 1.0 | 0.7 | 0.0 | 0.9 | 1.7 | U5 small nuclear ribonucleoprotein helicase | |
| VG1C153 | | | CU | | **3.7** | **20.5** | **23.2** | 1.4 | 1.9 | **12.7** |  |  | 0.5 | 0.5 | 0.9 | 0.1 | 1.7 | **3.8** | no hit | |
| VG1C050 | | | CU | | **3.5** | **24.1** | **26.1** | 1.7 | 1.9 | **30.2** |  |  | 0.5 | 1.1 | 1.0 | 0.1 | 2.4 | **4.8** | no hit | |
| NG1C125 | | | CU | | 1.1 | **34.3** | **24.0** | 2.2 | **3.6** | 34.8 |  |  | 0.5 | 1.9 | 1.1 | 0.1 | 2.0 | 1.7 | no hit | |
| Contig065 | | | CU | | 0.7 | **31.1** | **21.7** | 1.5 | **3.1** | **16.8** |  |  | 0.7 | 1.0 | 0.4 | 0.1 | 1.3 | 1.7 | 30 kDa seed maturataion protein (LEA) | |
| Contig061 | | | CU | | 1.1 | **22.9** | **18.5** | 1.1 | 1.8 | **7.9** |  |  | 0.5 | 0.7 | 0.6 | 0.1 | 1.0 | 1.2 | VrDhn1 | |
| Contig064 | | | CU | | 1.1 | **21.0** | **23.3** | 1.2 | **3.3** | **11.7** |  |  | 0.7 | 1.0 | 1.0 | 0.1 | 1.3 | 1.5 | polyadenylate-binding protein-interacting protein | |
| VG1C146 | | | CU | | 1.1 | **19.8** | **21.7** | 1.3 | 2.9 | **29.6** |  |  | 0.4 | 1.2 | 1.0 | 0.1 | 2.1 | 1.8 | no hit | |
| Contig056 | | | CU | | 1.1 | **19.6** | **20.2** | 1.1 | 2.4 | **8.0** |  |  | 0.6 | 0.5 | 0.6 | 0.1 | 1.0 | 1.5 | RPL10 | |
| NG3H034 | | | CU | | 1.2 | **15.5** | **12.1** | 1.2 | 2.2 | **8.8** |  |  | 0.6 | 1.3 | 0.9 | 0.2 | 1.3 | 1.3 | no hit | |
| VG1C253 | | | CU | | 2.1 | **14.4** | **13.5** | 0.8 | 1.3 | 28.8 |  |  | 0.6 | 0.8 | 0.1 | 0.1 | 2.7 | **32.9** | nuclease domain-containing protein | |
| Contig069 | | | CU | | 1.1 | **11.9** | **9.2** | 1.8 | 1.4 | **6.7** |  |  | 0.6 | 0.8 | 0.7 | 0.1 | 0.5 | 1.6 | no hit | |
| NG3H004 | | | CU | | 1.2 | **11.9** | **9.1** | 1.0 | 1.6 | **7.2** |  |  | 0.8 | 1.4 | 0.8 | 0.2 | 1.4 | 1.8 | glyoxalase II | |
| Contig115 | | | CU | | 1.8 | **11.7** | **10.1** | 0.6 | 1.0 | 36.0 |  |  | 1.1 | 0.9 | 0.1 | 0.1 | 1.9 | **30.4** | no hit | |
| VG3H087 | | | CU | | 2.0 | **8.9** | **12.9** | 0.9 | 1.4 | **17.3** |  |  | 1.1 | 0.7 | 0.2 | 0.1 | 2.4 | **34.9** | PDF2.3 | |
| Contig116 | | | CU | | 1.5 | **6.1** | **8.2** | 0.9 | 1.5 | **7.4** |  |  | 0.7 | 0.5 | 0.2 | 0.1 | 1.0 | **6.7** | no hit | |
| Contig063 | | | CU | | 1.1 | **5.4** | **5.3** | 1.1 | 1.7 | **4.6** |  |  | 0.6 | 0.6 | 0.9 | 0.2 | 1.0 | 1.5 | no hit | |
| NG3H027 | | | CU | | 1.4 | **4.0** | **6.7** | 0.8 | 1.1 | **5.1** |  |  | 0.7 | 0.4 | 0.3 | 0.2 | 0.8 | **6.6** | no hit | |
| Contig015 | | | CU | | 1.4 | **3.5** | **3.2** | 1.1 | 1.1 | **11.7** |  |  | 0.8 | 0.7 | 1.3 | 0.1 | 1.2 | 1.2 | 8S β-globulin | |
| VG3C101 | | | CU | | 1.6 | **3.5** | **5.0** | 1.1 | 1.4 | **3.1** |  |  | 0.9 | 0.6 | 0.5 | 0.3 | 1.2 | **4.2** | PDI | |
| VG1C154 | | | CU | | 1.1 | **3.5** | **3.3** | 1.2 | 1.3 | 2.4 |  |  | 0.7 | 0.7 | 0.7 | 0.3 | 0.8 | 1.1 | VHS and GAT domain protein | |
| VG3C226 | | | CU | | 1.1 | **3.4** | **3.9** | 0.9 | 1.1 | 2.7 |  |  | 0.5 | 0.5 | 0.8 | 0.4 | 1.0 | 1.5 | no hit | |
| Contig079 | | | CU | | 1.1 | **3.2** | **4.4** | 1.0 | 1.3 | 2.7 |  |  | 0.7 | 0.6 | 1.0 | 0.4 | 1.0 | 1.3 | 40S ribosomal protein S8 | |
| VG3C288 | | | CU | | 1.5 | **3.2** | **5.2** | 1.6 | 2.2 | 1.9 |  |  | 0.8 | 0.7 | 1.5 | 1.0 | 1.9 | 1.4 | no hit | |
| VG1C174 | | | LU | | 1.3 | 22.9 | **25.4** | 1.8 | 2.3 | 15.1 |  |  | 0.5 | 1.5 | 1.1 | 0.1 | 1.5 | 2.1 | no hit | |
| VG1C150 | | | LU | | 1.1 | 15.5 | **8.4** | 1.0 | 1.4 | **9.3** |  |  | 0.8 | 1.2 | 1.1 | 0.2 | 1.6 | 1.7 | BCCIP-like protein | |
| VG3C255 | | | LU | | 1.3 | 1.7 | **7.7** | 1.3 | 1.3 | 2.0 |  |  | 0.9 | 0.9 | 1.5 | 1.1 | 1.1 | 1.1 | transmembrane amino acid transporter | |
| VG3C021 | | | LU | | 0.5 | 2.4 | **4.4** | 0.9 | 0.8 | 0.2 |  |  | 0.4 | - | 1.7 | 1.2 | 0.6 | 0.4 | no hit | |
| MBB188 | | | LU | | 1.6 | 2.0 | **4.2** | 2.4 | **3.5** | 1.6 |  |  | 1.3 | 1.0 | 2.9 | 1.7 | 2.3 | 0.8 | Histone H3.3 | |
| VG3C235 | | | LU | | 1.3 | 1.1 | **4.2** | 2.7 | **3.2** | 1.8 |  |  | 0.9 | 1.0 | 2.4 | 2.4 | 2.0 | 1.1 | cold-regulated protein | |
| NG3C159 | | | \| LU \| \| --- \| \| LU \| \| LU \| \| LU \| \| LU \| \| LU \| | | 1.3 | 2.2 | **3.9** | 2.9 | **3.5** | 1.9 |  |  | 0.7 | 0.9 | 1.6 | 2.6 | 2.3 | 1.2 | no hit | |
| NG3C137 | | | LU | | 0.8 | 1.5 | **3.8** | 2.3 | 2.5 | 1.0 |  |  | 0.4 | 0.4 | 1.5 | 1.2 | 0.9 | 1.0 | no hit | |
| MBB151 | | | LU | | 0.9 | 1.4 | **3.7** | 2.6 | 2.2 | 1.9 |  |  | 0.6 | 0.6 | 2.5 | 1.7 | 2.2 | 1.6 | TIFY transcription factor | |
| NG3H017 | | | LU | | 1.2 | 1.8 | **3.4** | 0.7 | 0.9 | 1.8 |  |  | 0.4 | 0.4 | 0.4 | 0.3 | 0.6 | 1.6 | no hit | |
| Contig103 | | | LU | | 1.2 | 1.7 | **3.2** | 2.7 | 2.2 | 1.3 |  |  | 1.1 | 1.2 | 1.6 | 1.8 | 2.0 | 0.9 | cyclin-dependent kinase | |
| Contig123 | | | LU | | 1.0 | 1.2 | **3.2** | **3.3** | **3.3** | 1.5 |  |  | 0.6 | 0.5 | 1.1 | 1.9 | 1.7 | 1.7 | no hit | |
| VG3C092 | | | LU | | 1.4 | 4.1 | **3.1** | 1.0 | 1.27 | - |  |  | 0.6 | - | 0.8 | 0.6 | 1.4 | 1.2 | LETM1 and EF-hand domain-containing protein | |
| NG3H108 | | | LU | | 1.3 | 1.6 | **3.0** | 1.4 | 2.0 | 2.8 |  |  | 0.9 | 0.8 | 1.2 | 0.9 | 2.3 | 2.3 | no hit | |
| NG1C129 | | | LU | | 1.3 | 2.3 | 2.1 | **4.0** | **3.3** | 1.9 |  |  | 0.8 | 0.7 | 0.9 | 1.6 | 2.9 | 1.5 | NAC family protein | |
| VG1C268 | | | LU | | 1.5 | 2.5 | **3.1** | **4.1** | **11.7** | 5.5 |  |  | 0.6 | 0.4 | 1.27 | 0.7 | 0.9 | 2.2 | uncharacterized protein | |
| NG1C087 | | | LU | | 1.2 | 1.9 | 2.1 | 2.5 | **5.1** | 2.4 |  |  | 1.1 | 0.9 | 1.4 | 1.0 | 2.6 | **7.1** | thioredoxin (chloroplast) | |
| VG3H010 | | | LU | | 1.1 | 1.7 | 1.9 | 2.0 | **4.1** | 2.7 |  |  | 0.8 | 0.7 | 0.9 | 1.3 | 2.4 | 2.7 | no hit | |
| NG3C021 | | | LU | | 1.2 | 1.1 | 2.4 | 2.4 | **3.8** | 2.1 |  |  | 0.7 | 0.6 | 1.1 | 1.6 | 2.0 | 1.3 | protein GrpE | |
| NG3C287 | | | LU | | 0.8 | 1.2 | 2.3 | 1.7 | **3.0** | **3.9** |  |  | 0.5 | 0.4 | 0.7 | 0.8 | 1.4 | 2.8 | no hit | |
| Contig136 | | | ED | | 0.8 | **0.2** | 0.2 | 0.1 | 0.2 | 1.8 |  |  | 0.5 | 0.2 | 0.2 | 0.1 | 0.2 | 0.6 | xyloglucan endotraglucosylase/hydrolase | |
| MBA021 | | | LD | | 1.6 | 2.3 | 0.5 | **0.3** | **0.2** | 1.0 |  |  | 1.0 | 1.3 | 0.7 | 0.4 | 0.4 | 1.7 | UDP-D-apiose/UDP-D-xylose synthase 1 | |
| M056 | | | LD | | 1.2 | 1.8 | 0.8 | **0.3** | **0.3** | 1.1 |  |  | 1.1 | 1.1 | 0.8 | 0.4 | 0.3 | 1.3 | hypothetical protein | |
| **VC1973A *COR*s** | | | | | | | | | | | | | | | | | | | | |
| MBC247 | | | |  | 2.2 | 1.6 | 2.6 | 1.4 | 2.3 | **5.9** |  | LU | 0.8 | 0.9 | 2.1 | 0.8 | **4.8** | 2.3 | no hit | |
| MBA007 | | | |  | 1.1 | 1.2 | 1.6 | 1.7 | 2.8 | **8.4** |  | LU | 1.0 | 0.8 | 1.0 | 1. | **4.4** | **10.5** | no hit | |
| MBC171 | | | |  | 1.8 | 1.9 | 2.9 | 2.6 | 2.7 | 1.2 |  | LU | 1.2 | 1.1 | 2.8 | 2.4 | **4.3** | 0.8 | S-adenosylmethionine synthase | |
| Contig046 | | | |  | 2.8 | 1.9 | 2.4 | 1.9 | 2.7 | **9.5** |  | LU | 0.8 | 0.9 | 2.2 | 0.6 | **4.2** | 1.8 | SNF1-related protein kinase regulatory subunit β2 | |
| MBC157 | | | |  | 1.2 | 1.0 | 1.0 | 1.4 | 1.5 | 1.9 |  | LU | 1.0 | 0.8 | 1.4 | 1.8 | **3.4** | 1.8 | 30S ribosomal protein | |
| NG1C012 | | | |  | 1.7 | 2.0 | 2.3 | 0.5 | 1.1 | **4.3** |  | LU | 0.8 | 1.2 | 1.7 | 1.1 | **3.3** | **3.2** | ADH | |
| NG1C023 | | | |  | 1.2 | 1.4 | 1.7 | 1.5 | 1.6 | 1. 6 |  | LU | 1.5 | 1.7 | 2.0 | 1.6 | **3.1** | 0.9 | Cytochrome B561 | |
| Contig021 | | | |  | 0.9 | 1.3 | 2.1 | 1.6 | 2.3 | **4.0** |  | LU | 1.3 | 0.9 | 1.9 | 2.0 | **3.1** | 1.7 | Ribosomal protein S13 | |
| Contig057 | | | |  | 1.7 | 2.1 | 2.5 | 0.5 | 1.1 | **3.9** |  | LU | 0.9 | 1.2 | 1.7 | 1.1 | **3.1** | **3.5** | Uncharacterized protein | |
| ^a^ Fold change of each *COR* is the ratio of average intensity of chilling-treated to average intensity of the corresponding control. The bold indicates fold change ≥ 3 with a t test *p* value < 0.05. The underline of fold change at R3d represent *RCOR*. | | | | | | | | | | | | | | | | | |  |  |  |
| ^b^ ‘EU’ contains genes up-regulated by chilling only in 1-4 h; ‘LU’ contains genes up-regulated only in 24-72 h; ‘CU’ indicates genes up-regulated in 1-72 h; ‘ED’ contains genes down-regulated only in 1-4 h; ‘LD’ contains gene down-regulated only in 24-72 h. DP, dynamic pattern. | | | | | | | | | | | | | | | | | | |  |  |
